# PhantomAR: gamified mixed reality system for alleviating phantom limb pain in upper limb amputees—design, implementation, and clinical usability evaluation

**DOI:** 10.1186/s12984-025-01554-7

**Published:** 2025-02-04

**Authors:** Cosima Prahm, Korbinian Eckstein, Michael Bressler, Zhixing Wang, Xiaotong Li, Takashige Suzuki, Adrien Daigeler, Jonas Kolbenschlag, Hideaki Kuzuoka

**Affiliations:** 1 Department of Hand, Plastic, Reconstructive and Burn Surgery, University of Tübingen, BG Klinik, Tuebingen, Germany; 2 Department of Hand, Replantation and Microsurgery, Center for Musculoskeletal Surgery, Charité University Medicine, BG Unfallklinikum, Berlin, Germany; 3 School of Electrical Engineering and Computer Science, The University of Queensland, Brisbane, Australia; 4 Department of Mechano-Informatics, Graduate School of Information Science and Technology, The University of Tokyo, Tokyo, Japan

**Keywords:** Upper limb amputation, Hand amputation, Mirror therapy, Gamification, Phantom limb pain, Prosthesis, Myoelectric control, Mixed reality, Extended reality, Microsoft HoloLens 2

## Abstract

**Background:**

Phantom limb pain (PLP) is a restrictive condition in which patients perceive pain in a limb that is no longer present, greatly reducing their quality of life. Mirror Therapy, wherein patients observe a mirror reflection of their intact limb, has demonstrated efficacy in alleviating PLP. However, its unilateral and seated nature presents limitations. To address these constraints while still reducing PLP, and evaluating the impact of different virtual limb representations (anthropomorphic vs. non-anthropomorphic) on the user’s sense of ownership, agency, and embodiment, PhantomAR was developed. Leveraging wearable first-person augmented reality (AR) technology, PhantomAR extends traditional Mirror Therapy by enabling users to move freely and engage in bimanual tasks.

**Methods:**

The assistive mixed reality game application PhantomAR was deployed on the Microsoft HoloLens 2 and augmented the user’s residual limb by superimposing a virtual arm or tentacle that was controlled via residual muscles on their stump using an EMG electrode array. This setup allowed patients to engage in a first-person perspective and manipulate virtual objects with both the healthy and augmented limbs, free from the confines of a seated position. The study enrolled 10 able-bodied individuals and 8 individuals with unilateral, transradial amputation. All amputees experienced PLP. The usability of the PhantomAR application was evaluated using the System Usability Scale (SUS) and a user-centric survey. Additionally, the Game Experience was assessed on a 5-point Likert questionnaire (GEQ). Participants rated their phantom sensations using the Numerical Rating Scale and McGill Pain Questionnaire before, during, and after interaction with PhantomAR. The embodiment and agency of the virtual superimposed arm were evaluated with an altered Prosthesis Embodiment Scale. The study protocol included two sessions of 30 min each, during which participants experienced PhantomAR.

**Results:**

Participants (n = 18) rated PhantomAR highly usable (SUS m = 90.8%, SD = 6.88). Feedback on the Game Experience Questionnaire was overwhelmingly positive, showing high immersion (m = 4.46, SD = 0.08) and positive affect (m = 4.97, SD = 0.05). PLP (n = 8) significantly decreased post-intervention (NRS and McGill Pain Questionnaire, p < .001). Skin temperature in the residual limb increased significantly post-intervention (p < .01) but did not correlate with PLP (r = − 0.08, p = 0.83). Tentacle overlay yielded mixed ownership but high agency ratings.

**Conclusion:**

PhantomAR leverages mixed reality to significantly reduce Phantom Limb Pain, enhance user engagement, and alter perceptions of ownership and agency of their augmented limb through bi-manual, dynamic, full-body interactions.

*Trial registration* DRKS00033208 (Jan. 5th 2024)

**Supplementary Information:**

The online version contains supplementary material available at 10.1186/s12984-025-01554-7.

## Background

Many upper limb amputees report the sensation of a phantom limb, with some describing not only the presence but also various sensations associated with it. These sensations include proprioception of the phantom limb, awareness of its volume, spatial location, and occasional cramps or spasms [1, 2]. Additionally, an estimated 80% of amputees perceive painful sensations in their missing limb, referred to as Phantom Limb Pain (PLP) [3, 4]. It significantly diminishes their quality of life, causing distress and hindering daily activities, mental well-being, and overall health [3, 5–8]. PLP manifests in diverse ways, with common descriptions including burning, gnawing, lacerating, pressure, and distorted positioning [9–11]. Some patients experience improvement over time, while others may continue to have persistent pain, making it an issue that requires ongoing treatment [12, 13].

Efforts to manage PLP have encompassed both pharmacological and non-pharmacological approaches, but they often fall short of providing complete relief [8, 14–16]. The drawbacks of pharmacological treatments, such as potential side effects like daytime fatigue and personality changes, highlight the need for effective non-pharmacological alternatives. As for these, Mirror Therapy is the predominant treatment modality for Phantom Limb Pain. This therapy involves the placement of a mirror in a sagittal position adjacent to the patient’s intact limb, prompting the patient to visualize the reflection as a substitute for the contralateral amputated limb. This technique promotes a perception wherein the brain interprets the amputated limb as intact and mobile, effectively creating a non-painful illusion of the absent limb [17]. The efficacy of Mirror Therapy is largely attributed by neuroplasticity-based hypotheses of PLP to its provision of anthropomorphic visual feedback, which is recognized as a key factor in its therapeutic impact [18–20]. However, during Mirror Therapy, the patient is limited to only unilateral movements which, moreover, take place in a seated position. The patient does not have agency over the residual limb. These restrictive circumstances potentially limit the engagement, sustained motivation and embodiment of the patients, which are believed to be main driving factors of PLP reduction [19, 21–23].

Research has suggested that changes in skin temperature in the residual limb may correlate with the intensity of Phantom Limb Pain. Some studies have reported that increased pain intensity is associated with higher skin temperatures in the residual limb [24], while others have found no significant correlation [25, 26]. However, the amputation stump was almost always invariably colder than the corresponding point of the contralateral side [25, 27, 28]. Skin temperature is regulated by the body’s vasomotor response, which adjusts blood flow and consequently, skin temperature through processes like vasodilation and vasoconstriction [29]. Physical activity has been shown to boost circulation to the limbs [29], which could either contribute to pain perception in those with PLP or offer temporary relief by promoting relaxation and reducing muscle tension.

Recent advancements in PLP treatment increasingly leverage digital technologies such as Virtual Reality (VR), Augmented Reality (AR) and Mixed Reality (MR) using devices such as Meta Quest and Microsoft HoloLens to immerse users in virtual settings. In VR-based mirror therapy, the mirror image is substituted with a digital representation of the absent limb which is mirrored to the movements of the healthy limb [30–33]. AR extends this concept by superimposing virtual objects onto real-world views. This includes applications that project an augmented image of an intact limb over the residual limb on a computer screen using a camera and QR code [5, 34–37] or custom AR platforms by augmenting VR headsets with cameras to help alleviate Phantom Limb Pain (PLP) and train myoelectric control [38, 39]. Previous studies using screen-based AR primarily focused on myoelectric prosthesis control and the transferability of tasks from virtual environments to real-world settings, involving pick and place tasks [40] for pattern recognition control [41] or motor skill enhancement [42].

Mixed Reality advances this approach by allowing interactions between virtual and real objects, enhancing realism and engagement and spatial awareness, while first-person views via commercially available see-through glasses, such as the Microsoft HoloLens, Magic Leap or Google glasses, facilitate more accurate interactions and thus embodiment [43–51]. Immersive virtual reality technology is emerging as a successful nonpharmacologic adjunctive analgesic in reducing acute procedural pain. This is particularly evident in its application during dressing changes and in physical and occupational therapy [21].

In the context of healthcare gamification, research indicates that patient adherence to prescribed home rehabilitation exercises is often suboptimal, attributed to lack of motivation in absence of a supervising therapist [52, 53]. This challenge in motivation and compliance is a recurrent issue in clinical practice [54, 55]. Meta-analyses have highlighted the beneficial role of gamification strategies in enhancing health outcomes [56, 57]. Additionally, various studies have demonstrated the positive impact of gamification on therapy adherence, motivation, skill training, and learning in disease management [58–60]. A systematic review acknowledges the potential benefits of games for health while also underscoring the necessity for further methodologically sound studies in this [61]. Moreover, some researchers stress the importance of involving the target population, such as individuals with amputations, in the design and evaluation process. Observing how these users engage with mixed reality technology provides valuable insights into usability, user experience, and areas that may require adaptation or improvement to better meet their needs [62].

Building upon mirror therapy, PhantomAR offers an immersive mixed reality experience for individuals with transradial amputations. While mirror therapy utilizes visual illusion of a complete limb for pain reduction, PhantomAR extends this concept. It liberates patients from a static position, allowing free exploration and bi-manual interaction through a non-mirrored virtual limb. This virtual limb augments the residual limb and operates independently from their unaffected limb. Additionally, PhantomAR incorporates gamified elements to stimulate curiosity and engagement.

This study centered on designing, implementing, and evaluating PhantomAR, particularly focusing on:

- Evaluating the usability of PhantomAR, which allows free movement and bimanual interaction, and its effect on the intensity of PLP: We hypothesized that the intensity of PLP will decrease following the use of the mixed reality system. Additionally, we hypothesize that healthy participants will report high usability and engagement scores when using PhantomAR, with fewer physical challenges affecting their interaction, providing a baseline for system performance.
- Investigating the impact of different virtual limb representations (anthropomorphic vs. non-anthropomorphic) on ownership, agency, and embodiment: We hypothesized that there will be differences in the levels of ownership, agency, and embodiment experienced by able-bodied and amputated participants when interacting with anthropomorphic versus non-anthropomorphic virtual limb representations. However, we do not make a specific directional prediction, as the influence of non-traditional designs, such as a tentacle, remains largely unexplored.
- Exploring the potential relation between skin temperature changes and PLP: We hypothesized that increases in skin temperature will be observed during the use of PhantomAR, potentially correlating with a reduction in PLP. We further hypothesize that the magnitude of temperature changes will differ between the amputee and healthy group cohort.

## Methods

Participant recruitment for the study was conducted in compliance with the Declaration of Helsinki and followed the ethical guidelines by the University of Tuebingen, Germany (181/2020BO1). Prior to the initiation of the study, informed consent was obtained from all participants. Participants consisted of a cohort of ten able-bodied individuals (7 males, 3 females, aged 29.6 ± 8.6 years) and eight individuals with unilateral, transradial amputations (8 males, 2 females, aged 45.1 ± 7.8 years). Out of these 8 patients, 5 had already received a prosthesis, however, all stated that they did not use it regularly. All patients experienced medium to high PLP, which either appeared episodically (potentially triggered by activities or stress, with intermittent relief) or constant (mostly without significant periods of relief). 2 patients were taking regular pain medication (Table 1).

**Table 1:** Demographic data of the patients

Patient ID	Level of amputation	Stump length [% of intact limb]	Amputation side	Dominant hand	PLP Baseline	PLP frequency	Pain medication per day	Received prosthesis	Wears prosthesis	Received MT	MT worked	Age	Gender
1	TR	51%	Right	Right	7	Constant	None	y	n	y	n	45	f
2	TR	37%	Right	Right	4	Constant	None	y	y	y	n	57	m
3	TR	45%	Right	Right	5	Episodic	None	y	n	y	n	45	m
4	TR	47%	Right	right	4	Constant	None	y	y	n	-	37	m
5	TR	28%	right	right	7	Episodic	300mg GP	y	n	y	n	41	f
6	TC	80%	left	Right	5	Episodic	None	n	–	n	–	49	m
7	TR	42%	Left	Right	4	constant	200mg GP, 5 mg Oxycodone	n	–	n	–	33	m
8	TC	67%	Right	right	5	episodic	None	n	–	n	–	21	m

Stump length has been measured from the lateral epicondyles. MT = Mirror Therapy, GP = Gabapentin, y = yes, n = no

The usability of the PhantomAR application on the HoloLens 2 was assessed using the **System Usability Scale** (SUS). The SUS, a 10-item questionnaire using a 5-point Likert scale, is a widely accepted tool for evaluating a range of products, including software applications [63]. In addition, a **user-centric survey** comprising 10 questions was conducted to assess aspects such as immersion, ambience, control, interaction with virtual and real objects, and the comfort of wearing the HoloLens 2.

Participants’ motivation in using PhantomAR was evaluated using the **Game Experience Questionnaire (GEQ),** which includes 5 main subscales (positive affect, negative affect, flow, challenge, immersion) and 2 additional subscales for tentacle ownership and tentacle agency, rated on a 5-point Likert scale with 1 meaning "completely disagree" and 5 meaning "completely agree" [64].

Patients were additionally asked to rate their phantom limb pain before, during, and after the interaction using the Numerical Rating Scale (**NRS**). Phantom limb and sensation was further assessed by the German version of the Short Form McGill Pain Questionnaire (**SF-MPQ**) [65] during baseline and post-intervention measurements. The McGill Pain Rating Index (**PRI**) is constructed by adding up the scores of 15 pain qualities which are rated on a scale of 0 (“none”) to 3 (“severe”). Therefore, the PRI score ranges from 0 to 45.

**Skin temperature** was measured with a contactless infrared thermometer (MEM LEPU LFR30B) in the residual limb as well as in the uninjured limb of the amputee cohort and in the dominant hand used for playing of the able-bodied cohort, as it can be indicative of alterations in blood flow and muscle activity [28, 66, 67].

The embodiment and agency of the virtual superimposed arm/tentacle was evaluated using an altered **Prosthesis Embodiment Scale (PES)** by Bekrater-Bodmann [68], in which “prosthesis” was swapped out for “virtual arm”, and which consists of 10 items across 3 subscales for ownership (feeling as if the virtual arm belongs to oneself), agency (feeling in control of the virtual arm), and anatomical plausibility (the virtual arm being in an anatomically correct position relative to the user), with ratings from -3 (strongly disagree) to + 3 (strongly agree) [69, 70].

**Fig. 1:**
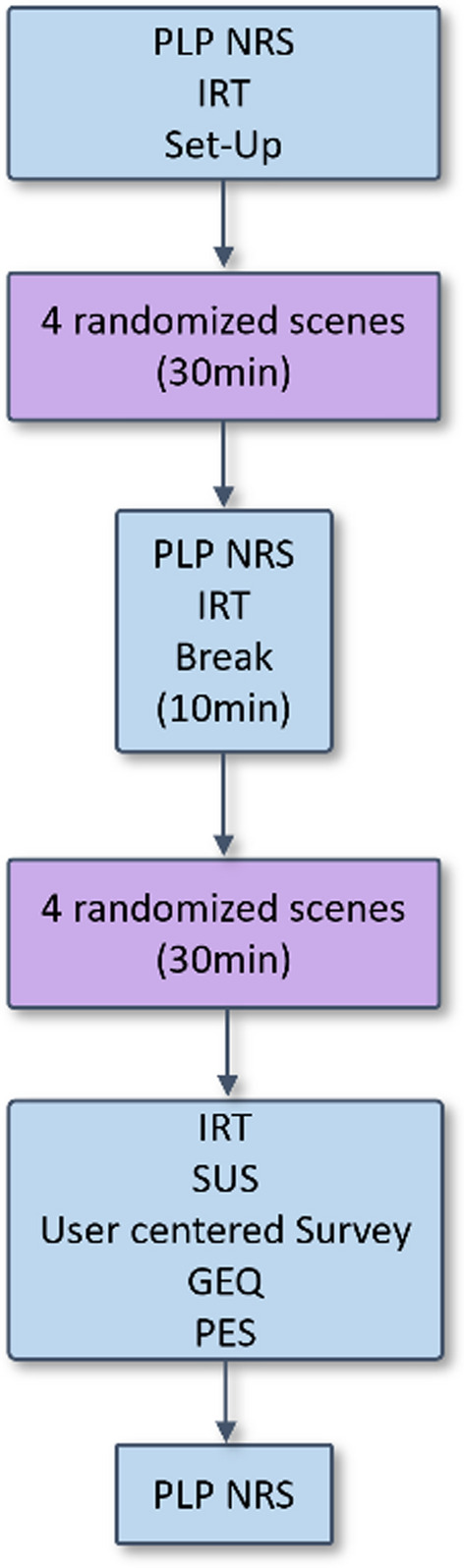
Flow-chart of the study protocol. After the first PLP questionnaire the Set-Up required under 5 min, including the donning of the devices and calibration of the virtual arm. (PLP = Phantom Limb Pain, NRS = Numerical Rating Scale, IRT infrared thermometer measurement, SUS = System Usability Scale, GEQ = Game Experience Questionnaire, PES = Prosthesis Embodiment Scale)

The usability evaluation of PhantomAR involved a single session of approximately 60 min being exposed to the application. The study protocol consisted of two sessions in which the participants were experiencing 4 randomized PhantomAR scenes, with the usability evaluation after both experiences and interleaved PLP NRS questionnaires (see Fig. 1). The real arm of able-bodied participants was obscured with a sleeve to prevent hand recognition by the HoloLens 2.

### Study setup

At the beginning of the study, participants sat comfortably in a chair in an examination room with ambient temperature of 22°C. Skin temperature was measured on the volar side of the stump and on the corresponding area on the contralateral, uninjured limb in patients and on the volar forearm in healthy participants. Patients were asked to rate their momentary PLP on the NRS scale.

**Fig. 2:**
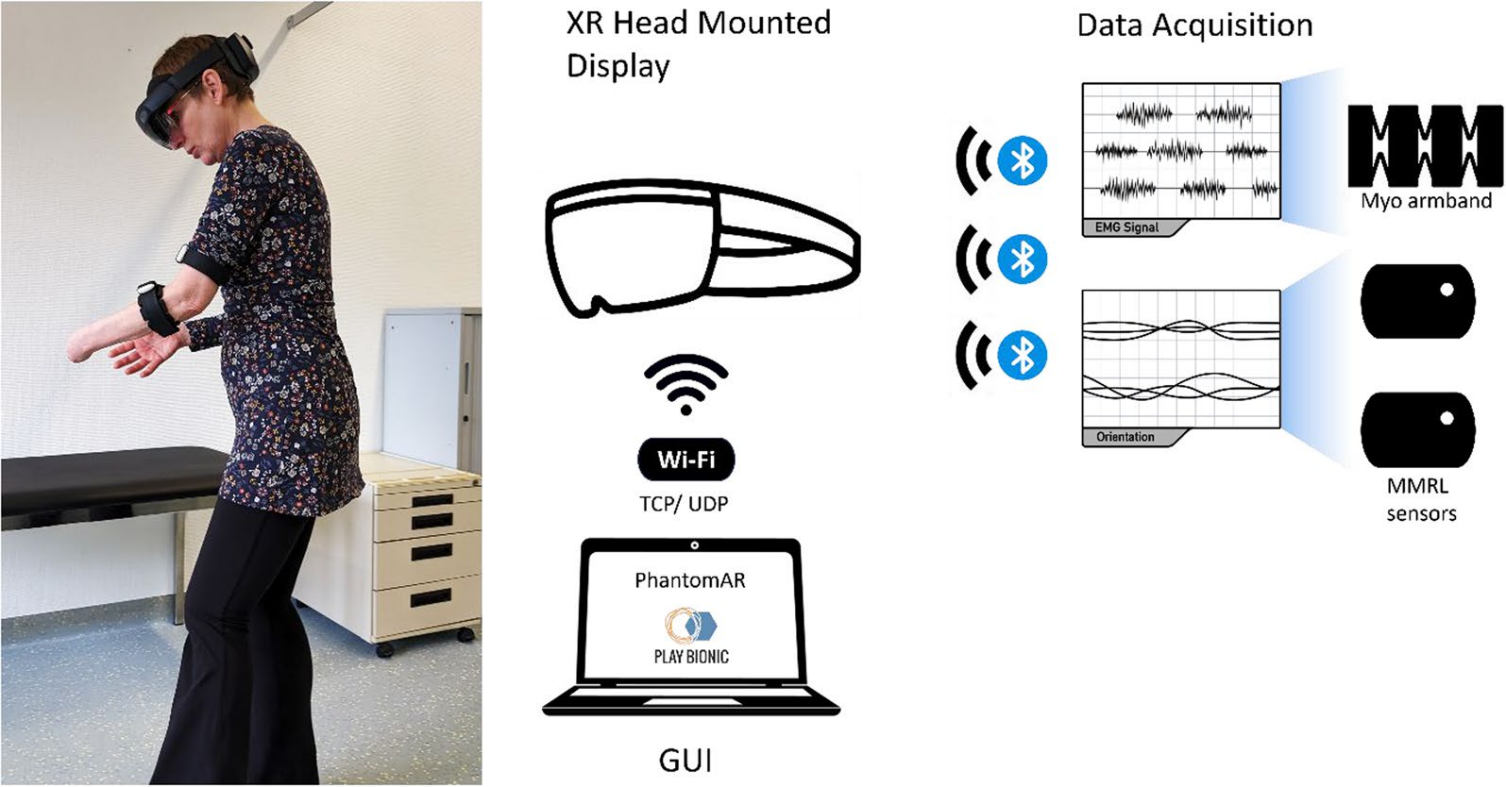
The PhantomAR system set-up consists of the extended reality device Microsoft HoloLens 2, the Thalmic Myo electrode armband which transmits EMG signals, and 2 MMRL sensors for orientation data of the superimposed digital arm. The set-up is completely wireless and does not restrict movement while patients can explore the virtual environments

The mixed reality study required a setup that was quick to implement for effective use in daily clinical practice. The equipment included a Microsoft HoloLens 2 headset on which the holograms of the mixed reality were projected, one Myo electrode armband (Thalmic Labs, Toronto, Canada, Note: discontinued by Thalmic Labs) and two Mbient Lab MMRL inertial measurement units (MBIENTLAB INC, San Jose, USA). The Myo armband featured 8 EMG electrodes, and a vibration motor for haptic feedback. MMRL sensors incorporated a 9-axis IMU. The setup was entirely wireless and battery-operated. Participants wore the Myo armband and an MMRL sensor on the residual lower limb and another MMRL sensor on the upper arm (see Fig. 2).

Interaction scenes within the application were designed to automatically adjust to available room sizes and shapes, ideally within 10–20 m^2^. Upon wearing the HoloLens 2, the virtual arm and myoelectric controls were calibrated, saving the user’s profile, including scale and relative shoulder position, in the app. This initial setup took under 5 min and was only necessary once.

Participants did not receive any further information beyond the essential instructions needed to operate and interact with the PhantomAR and were instructed simply to explore their environment which they did by walking around and interacting with various virtual and real elements.. Specifically, they were not provided with any details about the expected therapeutic effects on phantom sensations or pain. This approach was taken to minimize expectation bias and ensure that participants’ experiences and ratings were not influenced by preconceived notions about the intervention’s effectiveness.

After navigating the first four interaction scenes, patients were asked about their PLP. After playing another set of 4 random interaction scenes, participants evaluated the PhantomAR application, completing the SUS, the User centered survey, the altered PES and the GEQ. Their temperature was taken again at the same location as during the beginning of the trial and they were asked one last time about their momentarily PLP.

### Data analysis

Data were processed in Matlab version R2022b (Natick, Massachusetts: The MathWorks Inc). The Wilcoxon signed-rank test, as non-parametric test for related samples, was used for the following analyses: comparing medians of pre- and post-intervention NRS scores for PLP and the Short-Form McGill Pain Questionnaire (SF MPQ) Pain Rating Index (PRI); analyzing skin temperature changes in both the able-bodied and patient cohorts before and after the intervention; and comparing scores on the Embodiment Scale between amputees and able-bodied participants. The Mann–Whitney U test was used to determine differences between independent samples, such as the scores from the Game Experience Questionnaire (GEQ). Additionally, the Pearson correlation coefficient was employed to analyze the correlation between changes in skin temperature and PLP scores before and after the intervention. Given the small sample size, the level of statistical significance was consistently set at p < 0.01.

## Implementation

We designed and implemented 7 different environments for users to explore, as well as a separate graphical user interface for therapists and patients, using the game development platform Unity 3D and the Microsoft Mixed Reality Toolkit. The PhantomAR application was installed on the Microsoft HoloLens 2 and connected via Bluetooth to the Thalmic Myo electrode armband and the MMRL IMU sensors. The optional graphical user interface (GUI) for therapists was running on a laptop and connected to the PhantomAR HoloLens session via WI- FI.

### Game design and interaction scenes

The game design focused on immersion while minimizing mental stress, frustration and discomfort for patients, which are factors that could adversely affect PLP and the EMG control due to high muscle tension. Therefore, a curiosity driven gameplay was chosen, where the patients can freely explore an interesting and interactive environment without the possibility of failure or underperforming.

**Fig. 3:**
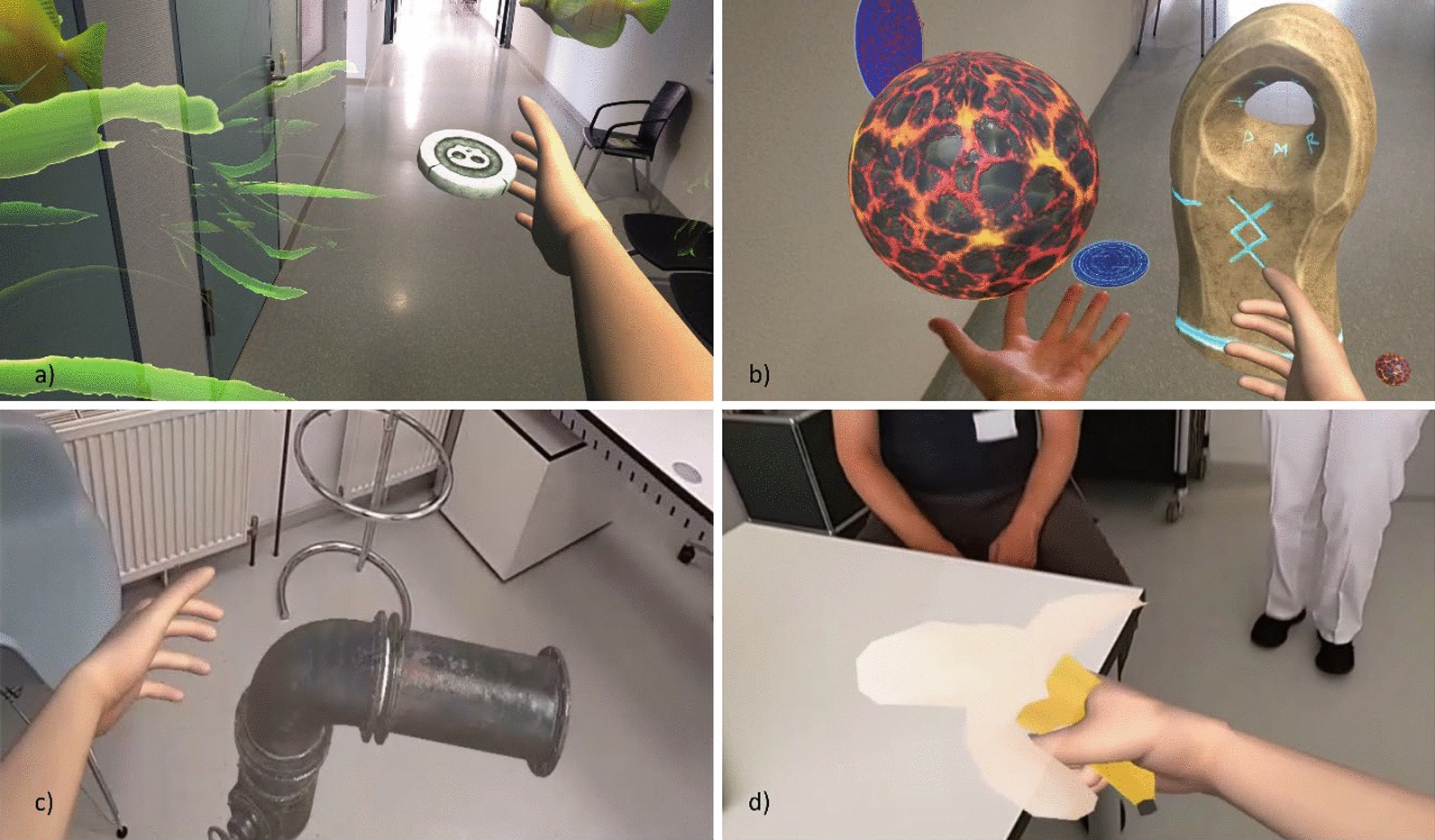
Screenshots of **a** Scene Aqua, which presents the player with various underwater elements to encourage interaction, i.e. the coin can be grabbed by the virtual or healthy hand and used to trigger certain events. **b** Scene Creature companion shows the interaction with a ball of lava that is bounced between the real and the virtual hand and can be evolved into a creature. The pillar is standing on the floor recognized by spatial recognition. **c** Pipes can be placed on walls, floor, or desks and interconnected. More pipe parts can be released via a virtual lever automatically placed on a wall. **d** Different fruits can be gathered and manipulated, such as resizing or squashing them

To allow patients to immerse themselves into the mixed reality experience, the rehabilitative exercises were integrated into various playful scenes (see Table 2). These scenes were designed without specific end goals, timers, or scoring systems. Instead, patients were encouraged to explore their surroundings with inquisitiveness, discovering the possibilities within each scene. This exploration involved touching, moving and resizing objects, interacting using one or both hands, and engaging with multiple objects simultaneously (see Fig. 3). The focus was on experiential learning and interaction rather than achieving specific objectives, fostering a more immersive and less pressured environment.

**Table 2:** Overview of the interactable scenes that were used in the study

Scene	Difficulty	Description	MyoControl
Aqua	3	Underwater simulation with interactive elements such as fish, algae, and corals integrated into the room’s spatial mapping environment. A treasure chest positions itself on any flat surface detected within the space. Participants are tasked with touching and combining virtual objects, which are designed to exhibit unexpected behaviors upon interaction	Interaction with both virtual and real hand simultaneously is possible, but not necessary
Liquid Creatures	3	Generation of sigils on the walls and a central virtual pillar on the actual floor. Participants interact with simulated liquids, eggs, and rune stones to initiate the spawning of various creatures within the space. Some of these virtual creatures are programmed to exhibit follower behavior, reacting to the participant’s movements	Interaction with both virtual and real hand simultaneously is possible, but not necessary
Pipes	2–3	Virtual levers are algorithmically generated on the walls of the environment. When these levers are activated by the participant, a virtual pipe appears, necessitating connection to an existing network of pipes through a series of grab-and-rotate interaction. Successful connections trigger the release of steam clouds	Interaction with both hands simultaneously is required
Space Music	2	Set within a virtual space environment, this scenario creates interaction with planets that produce musical outputs. Participants can create simple melodies by engaging with objects they place in the planet’s orbit	Interaction with both hands simultaneously is required
Fruit Picking	2	Collecting of virtual fruits that are algorithmically spawned within the environment, including less conspicuous locations such as under tables. The system allows participants to manipulate the size of these fruits by using a bilateral hand grip, enabling the fruits to fit into a designated collection basket	Interaction with both virtual and real hand simultaneously is possible, but not necessary
Drawing	1	Create three-dimensional drawings within the virtual space or on surfaces. This interaction is predominantly unilateral, with the augmented hand designated for the act of drawing. The selection of colors is managed by the contralateral hand, providing a more complementary role	Predominantly unilateral interaction with the virtual hand
Shooting	1	Participants aim and fire at target objects, represented by virtual flowers that are programmed to wilt and respawn upon being hit. The system enhances hand–eye coordination through the implementation of an aiming ray, guiding the participant’s actions	Predominantly unilateral interaction with the virtual hand

Each scene contains a distinct environment and follows different rules and interaction mechanisms to sustain patient engagement and curiosity. The interactive elements within each scene can be manipulated using both the virtual and the physical hand

### Spatial mapping

The HoloLens 2 supports automatic spatial mapping, enabling it to scan floors, walls, and tangible objects such as tables or smaller sized objects, allowing for the integration of virtual content with the real-world while adhering to height, width, or margin requirements. This feature is instrumental in making PhantomAR an environmentally aware application and supports various game mechanics, including the generation of plants on surfaces, interacting with floors, walls and ceilings, i.e. bouncing a ball on the floor, and placing virtual objects onto physical ones. However, Microsoft’s toolkit provided only basic surface data, which could occasionally extend beyond the current room. Therefore, a specialized wrapper layer was required to identify suitable object placement locations and improve game integration and responsiveness.

### Interaction with the virtual environment: arm and hand movements

Both the virtual and the intact hand were capable of interacting with virtual objects The residual limb was tracked using IMU data from two MMRL sensors positioned on the upper and lower arm. Combined with EMG data from a Thalmic Myo Armband on the residual lower arm, this setup enabled the creation of a virtual arm that the patient could freely control, much like a prosthesis. Previous horizontal drift over time could be addressed using these MMRL IMU sensors [47] instead of the Thalmic MyoArmband for spatial data. The position of the shoulder was fixed in relation to the head position and was adapted to match the individual user. In case the virtual arm should not align with the patient’s residual limb anymore, the virtual arm could be reset to the calibrated position with a light tap on the MyoArmband.

To enhance the naturalness of grasping movements with the virtual hand, we introduced auxiliary interaction mechanisms such as freezing the target object during grabbing and implementing a two-handed interaction for larger objects which necessitated the use of the contralateral healthy hand. The HoloLens 2 tracked the healthy hand, capturing the positions of the digits and palm, enabling them to interact with virtual objects and supporting rotating the object or resizing it. A short vibration from the Thalmic Myo armband accompanied successful grabs, reducing the time needed [42]. The virtual hand had attached colliders that closely matched its shape, enabling physical interactions with virtual objects, such as pushing a ball. Smaller objects could pass between the virtual fingers to create an immersive interaction experience.

Amputees controlled the virtual hand using a combination of IMU data from MMRL sensors and EMG signals from a Thalmic Myo Armband. The grasping action was initiated when muscle activation, recorded from two electrodes placed on antagonist/agonist muscles, exceeded a preset threshold. This EMG-based threshold controller allowed the virtual hand to open or close, with the speed of these actions being proportional to the muscle signal strength. When the patient activated the designated muscles above the threshold, the virtual hand closed; when the muscles relaxed or activated differently, the hand opened.

The control algorithm, however, is modularly adaptable and any controller, such as pattern recognition, can be easily integrated into the PhantomAR application and chosen via the GUI.

### Non-anthropomorphic feedback

**Fig. 4:**
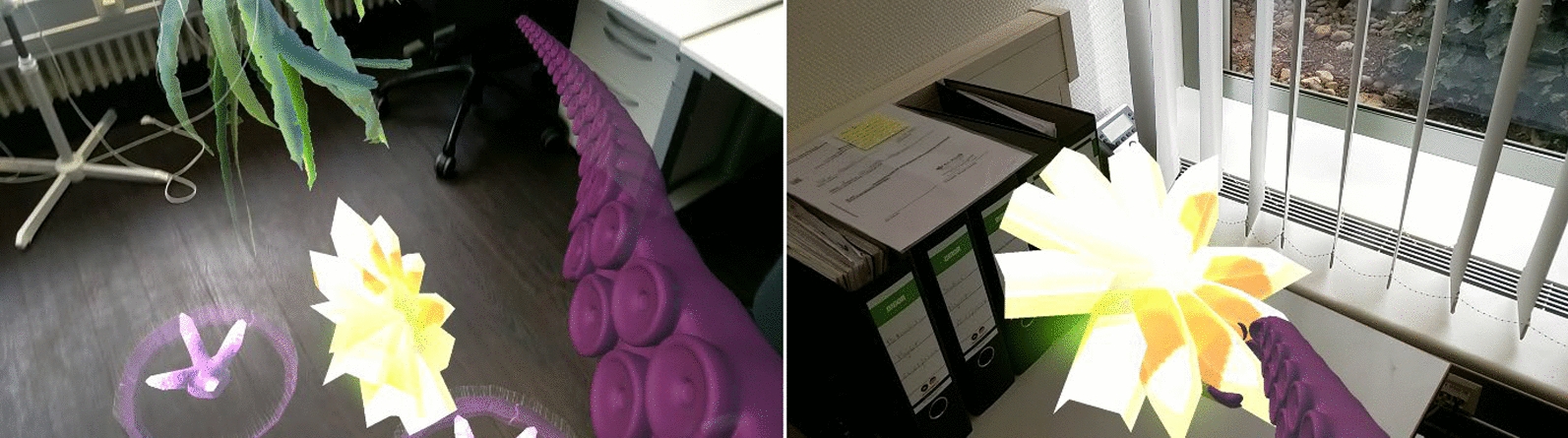
Tentacle extending and retracting according to the myoelectric signals from the user to grasp a virtual game object

Beyond just replicating a human arm, we allowed for the substitution of the arm model with a virtual tentacle (see Fig. 4) Both the arm and tentacle were controlled using the same motion range via EMG. However, instead of the hand’s opening and closing actions, the tentacle would extend and retract. Additionally, any wrist rotations or arm movements performed by the patients were correspondingly translated to the movements of the tentacle.

### GUI and remote connection for therapeutic supervision

**Fig. 5:**
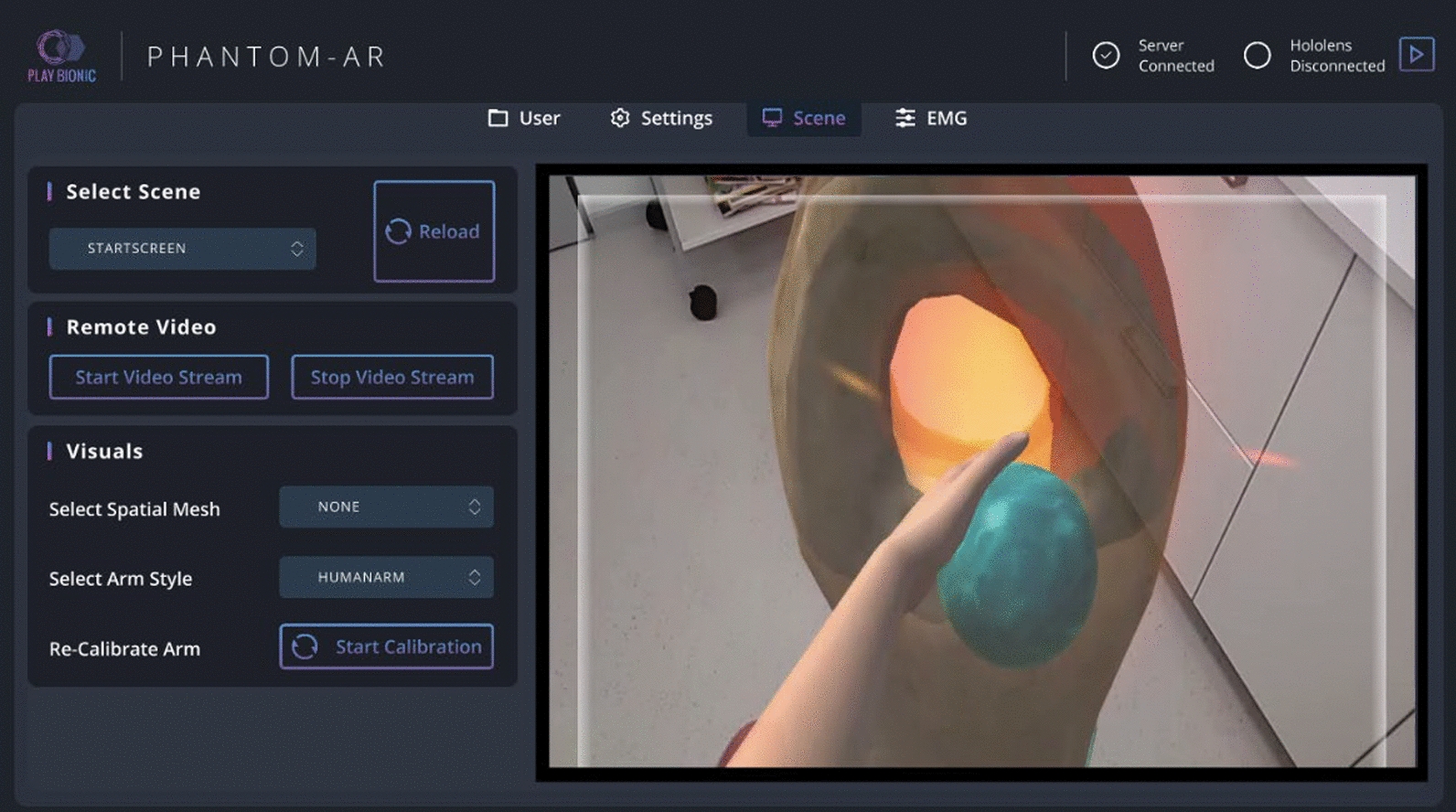
Remote connected GUI. The interface has different sections for managing the user data, settings, scene control and EMG calibration. In the selected Scene section, the left side provides control over the running session, while the live stream on the right side is used to monitor the patient experience. In the lower left corner of the live stream window, the patient’s stump can be seen, which is superimposed by the virtual arm

To facilitate therapist-led guidance and control over virtual scenarios, we developed a remote control application that operates on Microsoft Windows (Fig. 5). This optional app communicates with the HoloLens 2 via Wi-Fi, providing therapists a live video stream that mirrors the patient’s mixed reality view. It enhances versatility of the therapeutic process by enabling remote manipulation of virtual scenarios, such as manual creation or resetting of objects. Additionally, the GUI serves as a tool to simplify various configuration tasks, including Bluetooth connectivity setup, EMG controller calibration, and managing patient-specific parameters. However, everything can be adjusted within the HoloLens environment itself as well.

## Results

### System and game evaluation

The application received a System Usability Scale (SUS) score of 89.6% (SD = 6.9) by amputees and a score of 90.8% by able-bodied participants. This score reflects a high level of usability and user-friendliness, as all scores above 68% are considered above average [71].

**Fig. 6:**
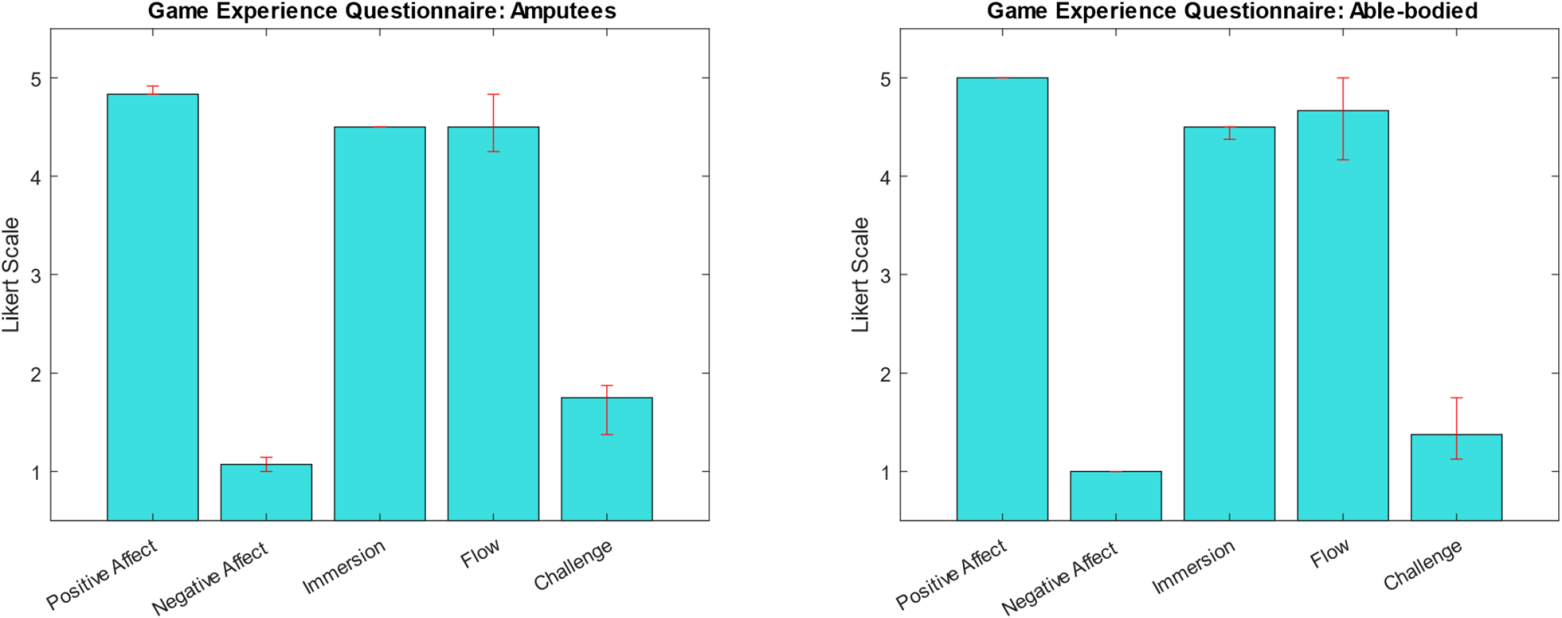
The median and IQR of the game experience questionnaire on a 5-point Likert scale as rated by patients (n = 8) and able-bodied participants (n = 10) show 5 subscales for positive and negative affect, immersion, flow

The Game Experience Questionnaire results for the patients and able-bodied participants are depicted in Fig. 6. The application received overwhelmingly positive feedback from all patients (md = 4.8, IQR = 0), with no reported negative emotions (md = 1, IQR = 0) or sensations of being overwhelmed by the challenge during gameplay (md = 1.75, IQR = 0.3). Additionally, both immersion (md = 4.5, IQR = 0) and game flow (md = 4.5, IQR = 0.3) received notably high ratings. The game experience for able-bodied participants was similar, with equal scores in the subscales positive affect (md = 5, IQR = 0), negative affect (md = 1, IQR = 0), immersion (md = 4.5, IQR = 0.18), flow (md = 4.5, IQR = 0.79). Challenge was rated slightly lower, but not significantly (md = 1.37, IQR = 0.68, p = 0.74) (see Fig. 6).

Prior mixed reality experience was limited, with 80% of able-bodied participants and all patients reporting no previous exposure.

### PLP and physical reaction

**Fig. 7:**
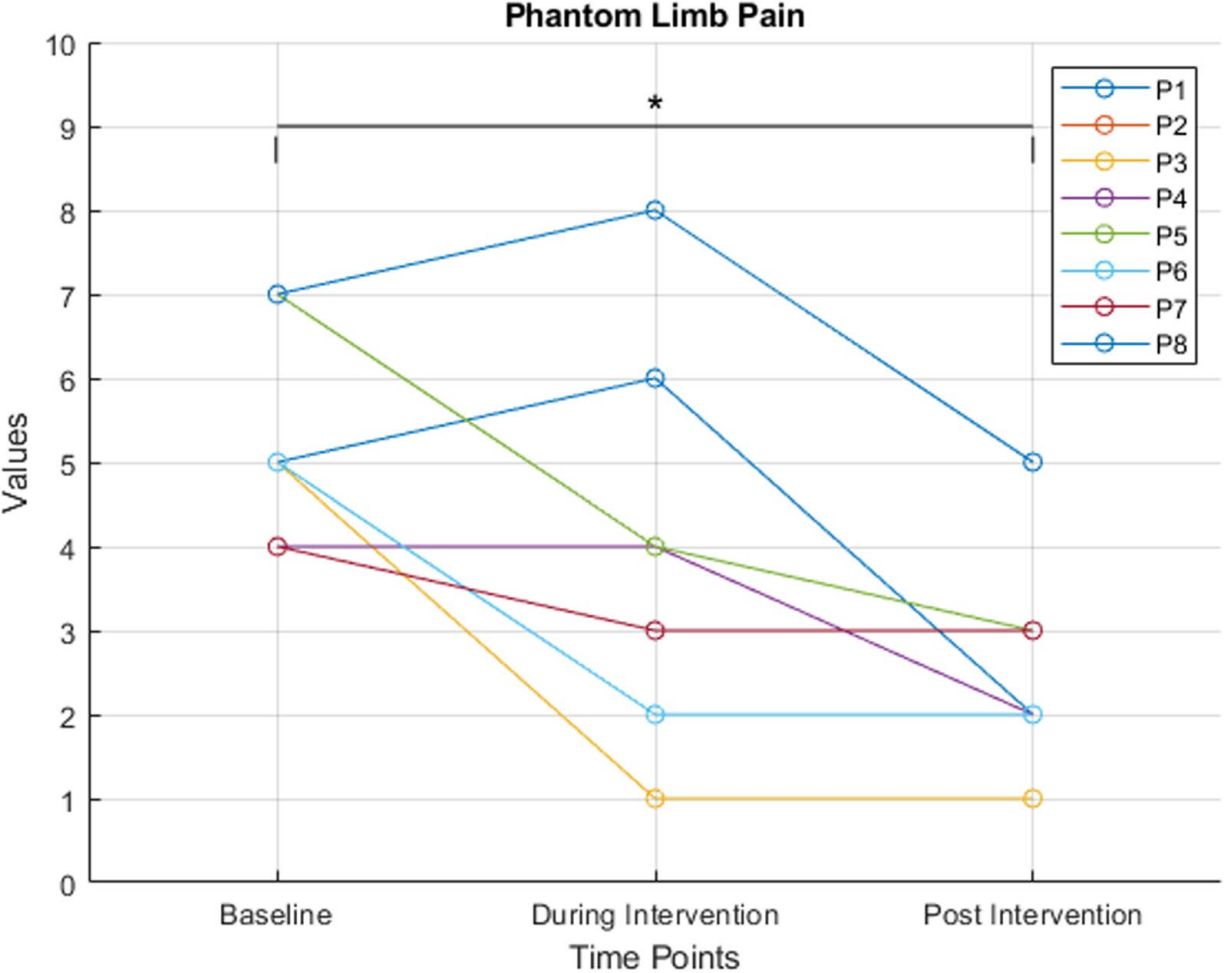
Progression of PLP (n = 8) as assessed with NRS showing a significant reduction indicated * p < 0.001 between the baseline and post-intervention measurement. High variance in PLP NRS score was observed both at the baseline and during the intervention

Initial PLP at baseline was rated with a median of 5 (IQR = 0.75) on the NRS scale by all patients before the intervention. When questioned about their PLP during gameplay, all participants reported a decrease in their PLP while immersed in the application. However, two patients reported an increase in pain, showing a high variance in PLP during the intervention (md = 3.5, IQR = 2.5). After finishing the application patients reported a significant decrease in PLP of approximately 58%, or a median of 3 points, respectively (p < 0.001), between the baseline and post-intervention measurement (md = 2, IQR = 1, see Fig. 7). Ranging from 25 to 80% reduction.

**Fig. 8:**
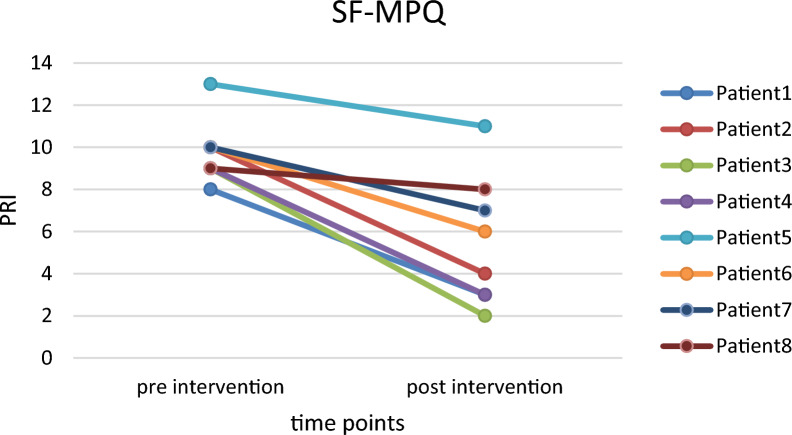
Pain Rating Index (PRI) of the patients’ PLP (n = 8) as assessed by the SF McGill questionnaire (SF-MPQ) showing a significant reduction from the baseline to after the PhantomAR experience (p < 0.01)

Similarly, pairwise comparisons of the SF-MPQ Pain Rating Index (PRI) scores revealed a significant reduction of approximately 45% from baseline to post-intervention (p < 0.001; see Fig. 8).

**Fig. 9:**
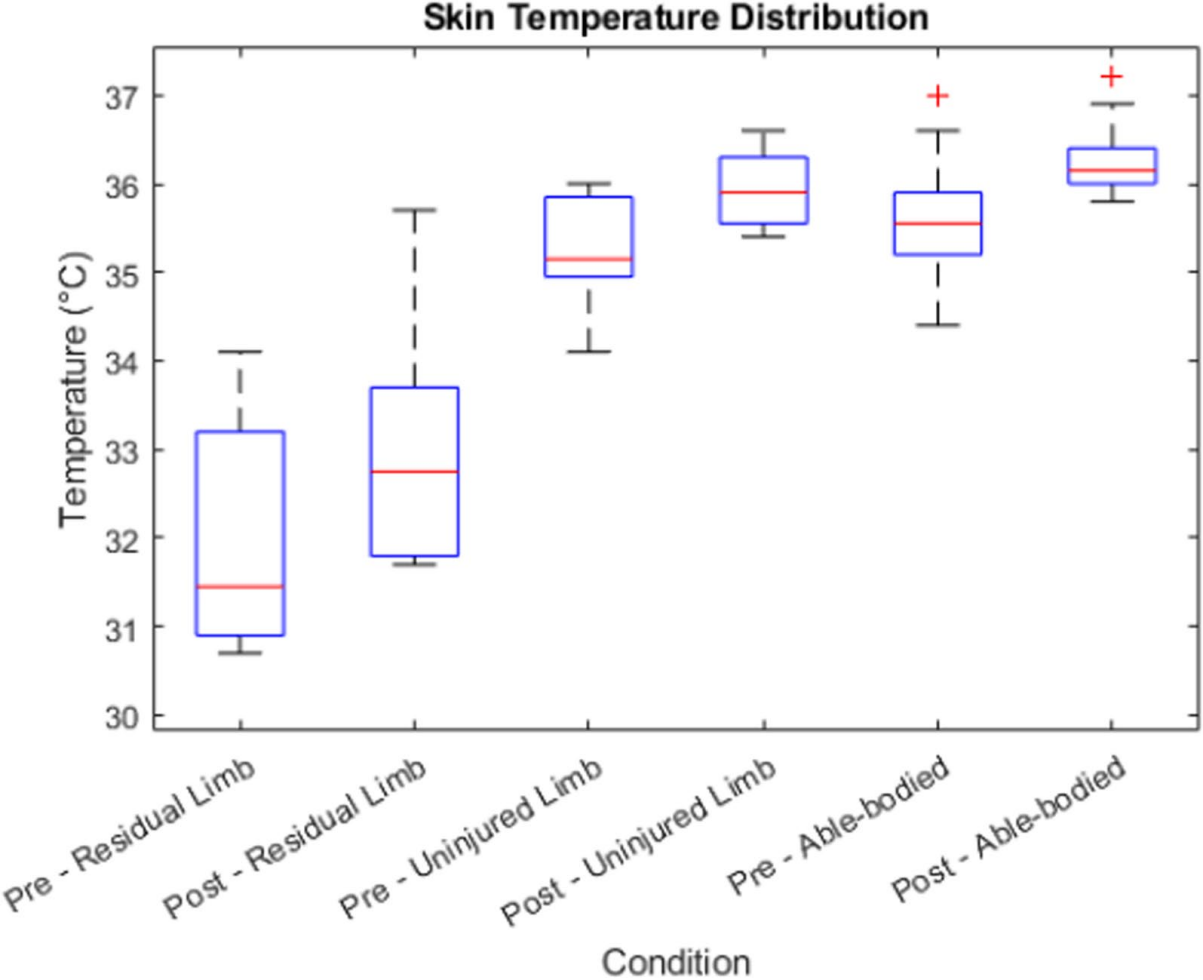
Skin temperature measured on the residual limb and the unaffected contralateral limb of patients (n = 8), and in able-bodied participants (n = 10) before and after the intervention, respectively

The skin temperature shows a significant increase from before the intervention to afterwards in all conditions as presented in Table 3, its distribution can be found in Fig. 9. The difference in skin temperature between the injured and unaffected arm was on average 3 °C, with the residual limb displaying colder temperatures, and temperature increased on average 1 °C in the residual limb over the course of the intervention. There was a high variance in temperature in the residual limb of patients that ranged from 30.7 °C to 34.1 °C before and from 31.7 °C to 35.7 °C after the intervention. There was no significant temperature difference between the patients’ uninjured arms and the arms of the able-bodied participants (p = 0.61).

**Table 3:** Skin temperature values in patient’s affected and contralateral healthy limb (n = 8) and in able-bodied participants (n = 10)

Condition	Mean temperature	Standard deviation	p
Pre—Residual Limb	31.9	1.33	< 0.01
Post—Residual Limb	32.9	1.37	
Pre—Healthy Limb	35.3	0.63	< 0.01
Post—Healthy Limb	35.9	0.44	
Pre—Able-bodied	35.6	0.76	< 0.01
Post—Able-bodied	36.2	0.45

A correlation analysis between PLP and the temperature of the residual limb before and after the intervention yielded a Pearson correlation coefficient of-0.09 and 0.19, respectively, indicating no significant linear relationship between these two variables (p = 0.83 and p = 0.64).

### Embodiment

**Fig. 10:**
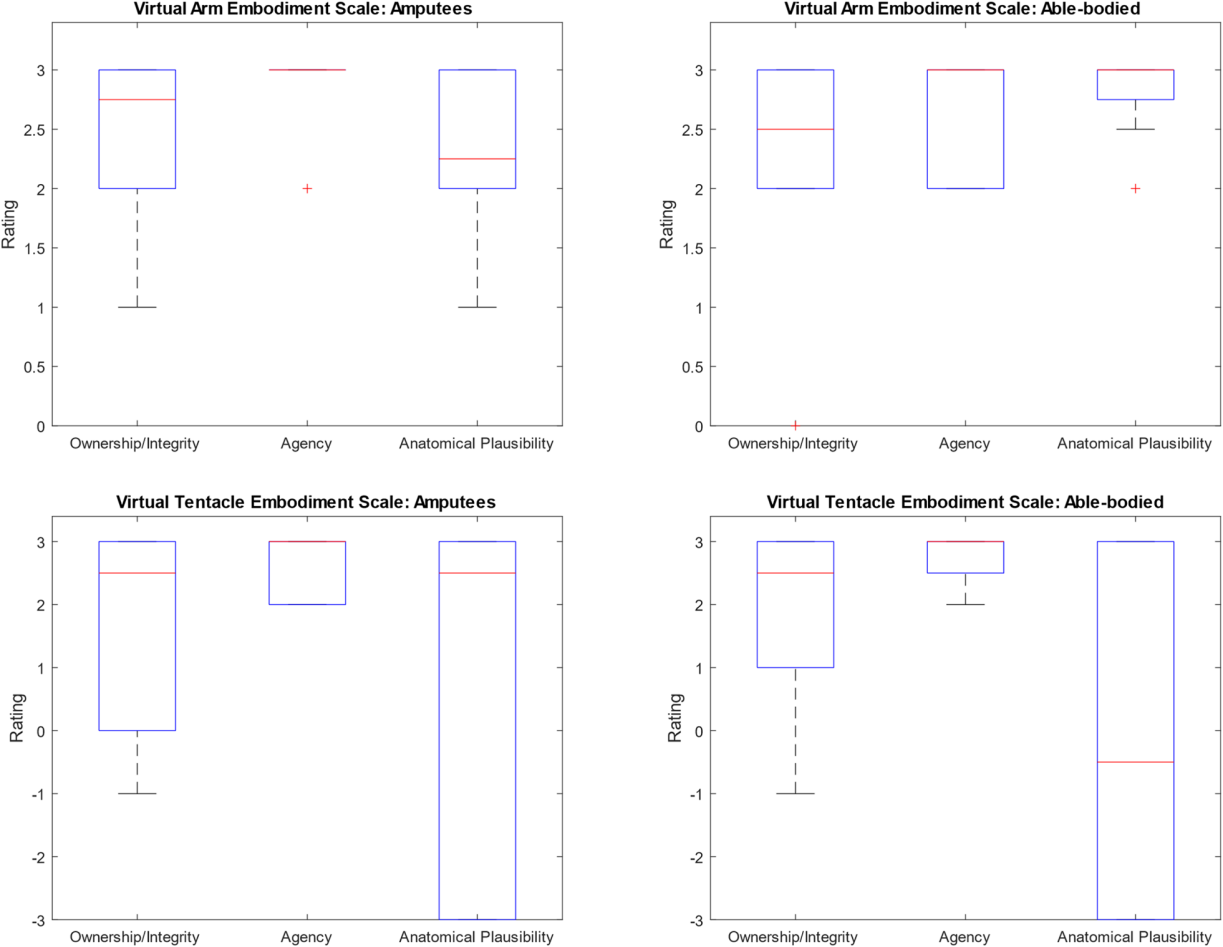
The 3 subscales of the adapted Prosthesis Embodiment Scale [− 3, + 3] for all patients (left side, n = 8 and all able-bodied participants (right side, n = 10). Questions about their prosthesis had been replaced by questions about the augmented virtual arm (upper row) or the augmented tentacle (lower row)

The modified Prosthesis Embodiment Scale [68], adapted to assess Virtual Arm Embodiment, revealed a high sense of agency among participants, indicating that participants felt cohesive control over the superimposed virtual arm and regarded the executed movements as their own (see Fig. 10).

Figure 10). Statistical analysis showed no significant differences in any of the subscales between healthy participants and amputees: Ownership/Integrity (p = 0.8), Agency (p = 0.5), and Anatomical Plausibility (p = 0.15).

### Anthropomorphic representation

The use of a tentacle overlay resulted in a moderate score and high variability in ownership scores among amputee participants (md = 2.5, IQR = 2). For able-bodied participants, tentacle ownership was rated similarly (md = 2.5, IQR = 2), with no statistically significant difference (p = 0.45). Tentacle agency was rated highly by amputee participants (md = 3, IQR = 0.5) and similarly high by able-bodied participants (md = 3, IQ = 0.5). However, the difference in agency scores between the two groups was also not significant (p = 0.8). Overall, both groups showed no significant differences in tentacle ownership or agency. Anatomical plausibility shows a high variance in both amputees (md = − 0.5, IQR = 6) and able-bodied participants (md = − 0.5, IQR = 6) as this item was hard to understand in this context of using a tentacle, as some reported the objective anatomical plausibility, and some compared it to a human arm (p = 0.8) (see Fig. 10).

According to the user centered survey, using a tentacle for a hand was a concept which was new to all patients, but they embraced the idea and stated, that it did not necessarily need to be their hand, or any hand for that matter. They reported it was fun to explore the PhantomAR application in real life and could see the room and their augmented arm. However, they preferred an anthropomorphic representation to a tentacle.

### User centered survey

Responses from all participants (n = 18) in the user-centered survey further revealed the following key observations: Users described wearing the HoloLens as comfortable, and though initially the field of view felt restricted, they soon forgot about it. They described interactions with virtual game elements in the real environment as novel, interesting and challenging, increasingly perceiving these objects as convincingly real. None reported experiencing cyber (motion) sickness during the study. The introduction of haptic feedback through the Thalmic Myo armband greatly enhanced the immersive experience of grasping objects, and participants found the controls to be intuitive.

Users commented that it was a long time since they could feel their hand and that they felt their phantom hand grow into the augmented hand. They said that they were surprised at how real everything looked and that they would like to just stay in this level (underwater level) and look around. There was not one comment that the HoloLens would be uncomfortable. When users “lost” their augmented arm, they could re-calibrate and would say “Ah, there is my hand again.”

## Discussion

With PhantomAR, we aimed to develop a wearable assistive therapy tool for Phantom Limb Pain (PLP) that extends traditional mirror therapy by liberating users from the restrictive seated position at a table and allowing for bimanual tasks and natural interaction with both virtual and actual objects. Addressing the complex phenomenon of PLP requires a flexible treatment approach, which PhantomAR provides through its modular design that accommodates several control methods [72]. PhantomAR was not designed to be goal-oriented, but curiosity driven. There is no intended or evaluated task transfer from a virtual hand to a myoelectric prosthesis.

### Mixed reality

By incorporating patient feedback into the design process, we ensured the clinical relevance of PhantomAR and addressed practical challenges that patients face. This user-centered approach has allowed us to optimize the system’s design for clinical use. The portable and wireless nature of PhantomAR makes it adaptable for various settings, from clinics to patients’ homes, with automated room detection enhancing ease of use. These features lay the groundwork for broader adoption in rehabilitation centers and at-home therapy.

Brasse et al. suggest, that mixed or augmented reality will play a significant role in future medical applications, enabling patients to perceive a fusion of virtual and real-world visuals [62]. By blending virtual projections with the real world, PhantomAR could serve as a bridge during the rehabilitation process. Specifically, it was designed to be used in the interim phase while the amputated limb is healing and before a permanent prosthetic is fitted, since using a prosthesis has been found to reduce PLP in most users [73].

### PLP

While our proof-of-concept study demonstrated promise, it is clear that a single-session design cannot fully capture the long-term impact of PhantomAR on PLP. We observed high variability in how patients experienced PLP during the intervention. For instance, some patients reported immediate pain relief during active use, only to note increased discomfort shortly thereafter, followed by eventual pain reduction, which all patients reported. Engaging in active, immersive tasks in augmented reality might divert attention from pain or activate neural pathways associated with motor control, which could modulate pain perception. The potential influence of distraction or cognitive load on pain reduction might be a valuable area to explore. Therefore, performing a long-term study while also increasing the sample size can not only provide more insight on PLP but also on embodiment over time.

We observed a 58% reduction in PLP on the NRS and a 45% reduction in the PRI during this single-session intervention. A clinically meaningful change in pain perception for amputees is typically defined as an NRS reduction of approximately 2 points on the NRS or 36% [74]. In comparison, Tilak et al. reported in a previous short-term study administering mirror therapy four times a pain reduction of 3.38 ± 2.33 on the NRS in 12 patients, demonstrating comparable efficacy to our single-session results [75]. However, most other studies involved interventions lasting 2 to 4 weeks and are therefore not directly comparable. For instance, Sumitani et al. (2008) observed a 30%–50% reduction in PLP in 11 out of 22 patients after mirror therapy [76], while Foell et al. (2014) reported a 27% reduction in PLP after 4 weeks of mirror therapy in a sample of 13 patients [17].

### Anthropomorphic representation

Our study’s exploration of different virtual limb representations, including an anthropomorphic arm and a non-anthropomorphic tentacle, was partially inspired by real-world experiences shared by amputees. Literature pertaining to neuroplastic hypotheses for alleviating PLP highlight the relevance of prioritizing anthropomorphic visual feedback [19, 77]. The concept of stochastic entanglement as hypothesized by Ortiz-Catalan, however, predicts that pain reduction would be independent of the level of anthropomorphic visual representation [18]. In our study, while agency was high, ownership did not receive a high score for the tentacle representation.

An additional consideration is the need to disentangle which aspects of our system drives the observed outcomes, particularly with respect to embodiment, agency, and the potential PLP reduction. One possibility is that the reduction in PLP relies heavily on the user’s ability to feel that the virtual limb truly belongs to them. If embodiment is essential, then only designs that closely resemble a human limb and are easily integrated into the user’s body schema would be effective. In this case, the anthropomorphic nature of the virtual limb would be a crucial factor in creating a successful therapeutic outcome. Another angle to consider is whether a sense of agency—feeling in control of the virtual limb—might be more important than embodiment. And the engaging, gamified aspects of the system might boost user involvement and overall effectiveness, regardless of the virtual limb’s anthropomorphism.

### Temperature

The increase in mean skin temperature in the post-condition phases, such as in the ‘Post Residual Limb’ and ‘Post Proband’ groups, could be indicative of increased blood flow to those areas. An elevation in skin temperature is often associated with vasodilation, where blood vessels widen to increase blood flow. This physiological response can be a result of various factors, including increased muscle activity. The difference in temperature between the residual limb and unaffected site of 3 °C corresponds to previous findings in upper and lower limb amputees alike [67, 78] and was expected, because stump vascularization is affected by amputation and the limited activity of the residual limb. Our findings indicate that elevated PLP scores prior to the intervention correlate with increased temperatures in the residual limb. In the context of rehabilitation or physical therapy, variations in skin temperature may serve as markers for enhanced blood flow or elevated muscle engagement, aligning with common objectives of these therapeutic interventions [79, 80].

In our study, the use of an augmented reality (AR) system inherently engages the sense of embodiment, as participants interact with a virtual limb that may feel like an extension of their body. This could mean that the temperature changes we observed might not be attributable to reductions in pain but could also be influenced by shifts in the participants’ sense of ownership and embodiment of the virtual limb [81].

### Bimanual interaction and myoelectric control

Although PhantomAR was not explicitly designed for myoelectric prosthesis training, its modular architecture makes it suitable for that purpose. By adapting to various control schemes, whether threshold-based or machine learning-driven, PhantomAR can support patients in preparing for prosthetic use.

The latency of the movement of the real arm to the visual representation of the corresponding virtual arm was not directly measured, but for arm movements, there is no noticeable lag. The latency is assumed to be below 50 ms, as the data is received from the MMRL sensors in real-time every 10 ms and translated to the virtual arm position within the next frame. A comparably low latency has not yet been reported in other studies, in which the latency was 500–800 ms when controlling a virtual arm using custom IMU sensors [42].

Immersion could be increased from a technical perspective by creating a spatially coherent experience of the virtual and real world that are responsively interacting with each other and underlying it with haptic feedback.

In a future study, to enhance the precision of arm tracking when rotating the head independently from the shoulders, a third MMRL sensor will be employed to monitor shoulder position, thereby creating a more accurate representation and adding additional Degrees of Freedom to the internal model of the patient’s arm, which could further improve agency and embodiment. Currently, PhantomAR is exclusively available for transradial (forearm) amputees, but in the future, we plan to extend it to transhumeral (upper arm) amputees as well.

### Limitations

The findings presented in this feasibility study are based on a single group analysis of 8 amputees. We acknowledge that the lack of a control group limits the strength of any conclusions regarding efficacy compared to conventional therapeutic approaches. A longitudinal study is needed to further investigate the findings. Reliance on self-reported measures for PLP intensity, embodiment, ownership, and agency might introduce a subjective bias. Consequently, the results and analyses should be considered in light of this. Moreover, the restricted field of view of the Microsoft HoloLens 2 could limit the immersive experience, especially when users move outside the central vision area.

## Conclusion

PhantomAR represents an innovative application in the treatment of Phantom Limb Pain, offering an immersive mixed reality experience that extends beyond the static limitations of traditional Mirror Therapy. By leveraging the capabilities of the HoloLens 2, PhantomAR enables amputees to engage in bimanual, full-body interactions with both anthropomorphic and non-anthropomorphic virtual limbs. The findings demonstrated a significant reduction in PLP following a single-session intervention, high usability, and an immersive experience that captivated users. Haptic feedback further enhanced the sense of immersion and realism, with users reporting a meaningful reconnection with their phantom limb. These preliminary findings emphasize the importance of developing flexible, user-centered, plug-and-play therapeutic tools for amputees. A longitudinal, controlled study is needed to validate these findings, assess the long-term impact of repeated interventions, and evaluate the sustained effects of PhantomAR on PLP, embodiment, and user engagement over time.

## Supplementary Information

Additional file 1.

Additional file 2.

## Data Availability

No datasets were generated or analysed during the current study.

## Abbreviations

GUI: Graphical user interface
GEQ: Game experience questionnaire
IMU: Inertial measurement unit
IRT: Infrared thermometer measurement
NRS: Numerical rating scale
PES: Prosthesis embodiment scale
PLP: Phantom limb pain
PRI: Pain rating index (SF McGill)
SUS: System usability scale
