# Supplementary material for: PhantomAR: gamified mixed reality system for alleviating phantom limb pain in upper limb amputees—design, implementation, and clinical usability evaluation
Source: J Neuroeng Rehabil. 2025 Feb 4;22:21. doi: 10.1186/s12984-025-01554-7 (PMC11796004; doi:10.1186/s12984-025-01554-7)
Supplement: Supplementary file 1 — Additional file 1. [file 12984_2025_1554_MOESM1_ESM.docx]

**User-centered survey questions**

1. Did you find wearing the Microsoft Hololens 2 on your head comfortable?
2. Did you experience any motion sickness/cyber sickness?
3. Did you notice the restricted field of view of the Hololens 2?
4. How intuitive did you find the control for the augmented hand for interacting with virtual objects - Were there any moments where you struggled to perform certain actions or gestures?
5. How intuitive did the bimanual tasks feel when interacting with objects using both your virtual and healthy hand?
6. How did the vibration feedback from the Myo Armband impact your interaction experience?
7. How easy was it to re-calibrate the virtual arm if you “lost” it during the use of PhantomAR?
8. How realistic and convincing did you find the virtual game elements when interacting with them in the real environment?
9. Which one was your favorite level?
10. How enjoyable did you find the overall experience with PhantomAR? What features or changes would you suggest to make the system even more enjoyable?
